# Diaqua­bis­{5-carb­oxy-2-[(1*H*-1,2,4-triazol-1-yl)meth­yl]-1*H*-imidazole-4-carboxyl­ato}zinc

**DOI:** 10.1107/S160053681104387X

**Published:** 2011-11-02

**Authors:** Jing-Hao Hao, Jian-Ling Wang

**Affiliations:** a Department of Quality Examination and Management, Zhengzhou College of Animal Husbandry Engineering, Zhengzhou, Henan 450011, People’s Republic of China

## Abstract

In the title compound, [Zn(C_8_H_6_N_5_O_4_)_2_(H_2_O)_2_], the six-coordinate Zn^II^ ion, which is located on an inversion center, has a distorted octa­hedral configuration. Each 5-carb­oxy-2-[(1*H*-1,2,4-triazol-1-yl)meth­yl]-1*H*-imidazole-4-carboxyl­ate ligand chelates to the Zn^II^ ion through a triazole N atom and a carboxyl­ate O atom in the equatorial plane. The coordination sphere is completed by two water mol­ecules in axial positions. There is an intra­molecular O—H⋯O hydrogen bond in the ligand. In the crystal, mol­ecules are linked *via* inter­molecular O—H⋯O, O—H⋯N and N—H⋯N hydrogen bonds, forming a three-dimensional structure.

## Related literature

For the assembly of multi-functional ligands with metal ions in the construction of two- and three-dimensional structures with special properties such as electrical conductivity, magnetism, host–guest chemistry, and catalysis, see: Eddaoudi *et al.* (2001 ▶). For metal complexes with N-containing ligands, such as 4,4-bipyridine and triazoles, see: Chang *et al.* (2010 ▶). For triazole derivatives complexed to Ru to form anti­tumor metal complexes, see: Komeda *et al.* (2002 ▶). For a silver(I) complex with a ligand containing both a carboxyl­ate and a triazole group, see: Xie *et al.* (2009 ▶). For the isostructural manganese(II) complex of the same ligand, see: Ding & Tong (2010 ▶).
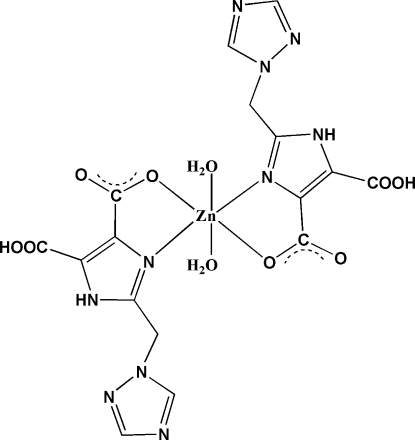


## Experimental

### 

#### Crystal data

[Zn(C_8_H_6_N_5_O_4_)_2_(H_2_O)_2_]
*M* *_r_* = 573.76
Monoclinic, 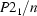

*a* = 7.7020 (15) Å
*b* = 14.678 (3) Å
*c* = 9.2912 (19) Å
β = 96.22 (3)°
*V* = 1044.2 (4) Å^3^
*Z* = 2
Mo *K*α radiation
μ = 1.26 mm^−1^
*T* = 293 K
0.15 × 0.15 × 0.10 mm

#### Data collection

Rigaku Mercury CCD diffractometer
Absorption correction: multi-scan (*CrystalClear*; Rigaku, 2000 ▶) *T*_min_ = 0.834, *T*_max_ = 0.884
8121 measured reflections
2048 independent reflections
1783 reflections with *I* > 2σ(*I*)
*R*_int_ = 0.038

#### Refinement

*R*[*F* ^2^ > 2σ(*F* ^2^)] = 0.045
*wR*(*F* ^2^) = 0.095
*S* = 1.11
2048 reflections
177 parameters
H atoms treated by a mixture of independent and constrained refinement
Δρ_max_ = 0.31 e Å^−3^
Δρ_min_ = −0.28 e Å^−3^

### 

Data collection: *CrystalClear* (Rigaku, 2000 ▶); cell refinement: *CrystalClear*; data reduction: *CrystalClear*; program(s) used to solve structure: *SHELXS97* (Sheldrick, 2008 ▶); program(s) used to refine structure: *SHELXL97* (Sheldrick, 2008 ▶); molecular graphics: *SHELXTL* (Sheldrick, 2008 ▶); software used to prepare material for publication: *SHELXTL*.

## Supplementary Material

Crystal structure: contains datablock(s) global, I. DOI: 10.1107/S160053681104387X/su2331sup1.cif

Structure factors: contains datablock(s) I. DOI: 10.1107/S160053681104387X/su2331Isup2.hkl

Additional supplementary materials:  crystallographic information; 3D view; checkCIF report

## Figures and Tables

**Table 1:** Hydrogen-bond geometry (Å, °)

*D*—H⋯*A*	*D*—H	H⋯*A*	*D*⋯*A*	*D*—H⋯*A*
N5—H5*A*⋯N3^i^	0.86	1.95	2.801 (4)	170
O3—H3*C*⋯O2	0.85	1.65	2.493 (3)	173
O5—H5*B*⋯O4^ii^	0.73 (5)	2.04 (5)	2.764 (4)	168 (5)
O5—H5*C*⋯N2^iii^	0.78 (5)	2.23 (5)	2.896 (4)	143 (5)

Symmetry codes: (i) 
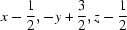
; (ii) 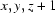; (iii) 
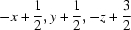
.

**Fig. 1.**
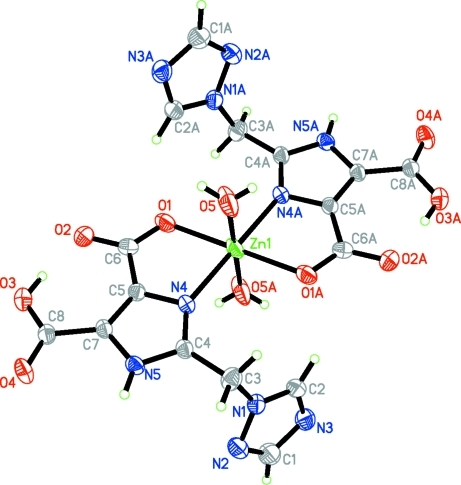
The molecular structure of the title compound, showing 50% probability displacement ellipsoids and the crystallographic numbering scheme [symmetry code: (A) 1 - x, 2 - y, 2 - z].

## supplementary crystallographic information

### Comment

The assembly of multifunctional ligands with metal ions is currently of great interest due to their use in constructing two- and three-dimensional compounds with special properties (Eddaoudi *et al.*, 2001). So far, most of these multi-dimensional coordination compounds are formed with N-containing ligands, such as, 4,4-bipyridine, polycarboxylic, and triazoles (Chang *et al.*, 2010). Triazole derivatives have been studied as anti-inflammatory drug candidates and have also been used as ligands for binding Pt and Ru to form antitumor metal complexes (Komeda *et al.*, 2002).

A system taking advantage of the presence of both a carboxylate and a triazol group for coordintaion to silver(I) has been reported on by (Xie *et al.*, 2009). The isostructural manganese(II) complex of the title ligand, 2-(1*H*-1,2,4-triazol-1-yl)methyl]-1*H*- imidazole-4,5-dicarboxylic acid, has been reported on by (Ding & Tong, 2010). Herein, we report on the synthesis and crystal structure of the title zinc(II) complex.

In the title compound, the zinc^II^ atom is located on an inversion center and is six-coordinated, by two imidazole nitrogen atoms (N4 and N4A) and two carboxylate oxygen atoms (O1 and O1A) of two deprotonated 2-((1H-1,2,4-triazol-1-yl)methyl)-1H-imidazole-4,5-dicarboxylic acid ligands, in the equitorial plane, and by two water molecules in axial positions (Fig. 1). The coordination Zn–N bond lengths are 2.110 (2) Å, while the Zn—O bond lengths are 2.115 (2) Å in the equitorial plane and 2.154 (3) Å in axial positions. The coordination geometry around the Zn^II^ ion can be described as distorted octahedral because the O–Zn–N and O–Zn–O coordination angles range from 79.48 (8)° to 100.52 (8)°. There is an intramolecular O—H···O hydrogen bond in each ligand (Table 1). This geometry is very smilar to that in the isostructural manganese(II) complex mentioned above.

In the crystal, molecules are linked *via* intermolecular O—H···O, O—H···N and N—H···N hydrogen-bonds, to form a three-dimensional structure (Table 1).

### Experimental

For the synthesis of title compound, a solution of [2-(1*H*-1,2,4-triazol-1-yl)methyl]-1*H*- imidazole-4,5-dicarboxylic acid)(1.0 mmol), Zn(NO_3_)_2_.6H_2_O (0.5 mmol) and NaOH (0.1 mmol) in 10 ml water was stirred for 30 min and then filtered. The filtrate was left to evaporate slowly at room temperature. After two days colourless single crystals, suitable for X-ray analysis, were obtained. Anal. Calcd(%) for C_16_H_16_ZnN_10_O_10_: C, 33.49; H, 2.81; N, 24.41. Found: C, 33.22; H, 2.60; N, 25.50.

### Refinement

The water H atoms were located in difference Fourier maps and were freely refined. The remaining H atoms were fixed geometrically and treated as riding atoms: O–H = 0.85 Å, N–H = 0.86 Å, and C–H = 0.93 and 0.97 Å for CH and CH_2_ atoms, respectively, with U_iso_(H) = 1.2U_eq_(parent O, N, or C atom).

### Crystal data

**Table:**

[Zn(C_8_H_6_N_5_O_4_)_2_(H_2_O)_2_]	*F*(000) = 584
*M**_r_* = 573.76	*D*_x_ = 1.825 Mg m^−^^3^
Monoclinic, *P*2_1_/*n*	Mo *K*α radiation, λ = 0.71073 Å
Hall symbol: -P 2yn	Cell parameters from 2869 reflections
*a* = 7.7020 (15) Å	θ = 2.2–30.8°
*b* = 14.678 (3) Å	µ = 1.26 mm^−^^1^
*c* = 9.2912 (19) Å	*T* = 293 K
β = 96.22 (3)°	Blocky, colourless
*V* = 1044.2 (4) Å^3^	0.15 × 0.15 × 0.10 mm
*Z* = 2	

### Data collection

**Table:**

Rigaku Mercury CCD diffractometer	2048 independent reflections
Radiation source: fine-focus sealed tube	1783 reflections with *I* > 2σ(*I*)
graphite	*R*_int_ = 0.038
ω scans	θ_max_ = 26.0°, θ_min_ = 2.6°
Absorption correction: multi-scan (*CrystalClear*; Rigaku, 2000)	*h* = −9→8
*T*_min_ = 0.834, *T*_max_ = 0.884	*k* = −18→18
8121 measured reflections	*l* = −11→11

### Refinement

**Table:**

Refinement on *F*^2^	Primary atom site location: structure-invariant direct methods
Least-squares matrix: full	Secondary atom site location: difference Fourier map
*R*[*F*^2^ > 2σ(*F*^2^)] = 0.045	Hydrogen site location: inferred from neighbouring sites
*wR*(*F*^2^) = 0.095	H atoms treated by a mixture of independent and constrained refinement
*S* = 1.11	*w* = 1/[σ^2^(*F*_o_^2^) + (0.0395*P*)^2^ + 0.6397*P*] where *P* = (*F*_o_^2^ + 2*F*_c_^2^)/3
2048 reflections	(Δ/σ)_max_ < 0.001
177 parameters	Δρ_max_ = 0.31 e Å^−^^3^
0 restraints	Δρ_min_ = −0.28 e Å^−^^3^

### Special details

**Table:**

Geometry. All e.s.d.'s (except the e.s.d. in the dihedral angle between two l.s. planes) are estimated using the full covariance matrix. The cell e.s.d.'s are taken into account individually in the estimation of e.s.d.'s in distances, angles and torsion angles; correlations between e.s.d.'s in cell parameters are only used when they are defined by crystal symmetry. An approximate (isotropic) treatment of cell e.s.d.'s is used for estimating e.s.d.'s involving l.s. planes.
Refinement. Refinement of *F*^2^ against ALL reflections. The weighted *R*-factor *wR* and goodness of fit *S* are based on *F*^2^, conventional *R*-factors *R* are based on *F*, with *F* set to zero for negative *F*^2^. The threshold expression of *F*^2^ > σ(*F*^2^) is used only for calculating *R*-factors(gt) *etc*. and is not relevant to the choice of reflections for refinement. *R*-factors based on *F*^2^ are statistically about twice as large as those based on *F*, and *R*- factors based on ALL data will be even larger.

### Fractional atomic coordinates and isotropic or equivalent isotropic displacement parameters (Å^2^)

**Table:**

	*x*	*y*	*z*	*U*_iso_*/*U*_eq_	
C1	0.4693 (4)	0.6509 (2)	0.7392 (3)	0.0374 (8)	
H1	0.5221	0.6068	0.6867	0.045*	
C2	0.4094 (4)	0.7314 (2)	0.9126 (3)	0.0335 (7)	
H2	0.4072	0.7573	1.0037	0.040*	
C3	0.1835 (4)	0.8324 (2)	0.7795 (3)	0.0324 (7)	
H3A	0.1465	0.8456	0.8738	0.039*	
H3B	0.0816	0.8131	0.7164	0.039*	
C4	0.2560 (4)	0.91683 (19)	0.7197 (3)	0.0238 (6)	
C5	0.4032 (4)	1.03992 (19)	0.6977 (3)	0.0227 (6)	
C6	0.5302 (4)	1.1118 (2)	0.7490 (3)	0.0270 (6)	
C7	0.3112 (4)	1.02311 (19)	0.5658 (3)	0.0223 (6)	
C8	0.2953 (4)	1.0733 (2)	0.4267 (3)	0.0252 (6)	
N1	0.3107 (3)	0.75838 (17)	0.7936 (3)	0.0283 (6)	
N2	0.3478 (3)	0.70673 (18)	0.6789 (3)	0.0352 (6)	
N3	0.5109 (3)	0.66274 (18)	0.8832 (3)	0.0348 (6)	
N4	0.3662 (3)	0.97318 (16)	0.7941 (2)	0.0242 (5)	
N5	0.2194 (3)	0.94454 (16)	0.5827 (2)	0.0250 (5)	
H5A	0.1505	0.9178	0.5168	0.030*	
O1	0.5950 (3)	1.10733 (15)	0.8783 (2)	0.0345 (5)	
O2	0.5641 (3)	1.17290 (14)	0.6601 (2)	0.0338 (5)	
O3	0.3923 (3)	1.14527 (14)	0.4209 (2)	0.0350 (5)	
H3C	0.4537	1.1587	0.4996	0.042*	
O4	0.1948 (3)	1.04696 (16)	0.3247 (2)	0.0363 (5)	
O5	0.2866 (4)	1.0846 (2)	1.0514 (3)	0.0512 (8)	
H5B	0.266 (6)	1.082 (3)	1.127 (5)	0.081 (18)*	
H5C	0.272 (6)	1.133 (3)	1.017 (5)	0.080 (18)*	
Zn1	0.5000	1.0000	1.0000	0.03113 (18)	

### Atomic displacement parameters (Å^2^)

**Table:**

	*U*^11^	*U*^22^	*U*^33^	*U*^12^	*U*^13^	*U*^23^
C1	0.0402 (19)	0.0351 (19)	0.0368 (19)	0.0051 (15)	0.0033 (15)	−0.0006 (14)
C2	0.0437 (19)	0.0294 (17)	0.0254 (16)	−0.0050 (14)	−0.0058 (13)	−0.0005 (13)
C3	0.0290 (16)	0.0310 (17)	0.0362 (18)	−0.0006 (13)	−0.0008 (13)	0.0056 (13)
C4	0.0250 (14)	0.0256 (15)	0.0203 (14)	0.0018 (12)	0.0004 (11)	0.0021 (11)
C5	0.0253 (15)	0.0226 (14)	0.0208 (14)	0.0033 (12)	0.0054 (11)	0.0002 (11)
C6	0.0279 (16)	0.0260 (16)	0.0273 (16)	0.0006 (12)	0.0040 (12)	−0.0058 (12)
C7	0.0236 (14)	0.0243 (15)	0.0189 (14)	0.0034 (11)	0.0020 (11)	0.0014 (11)
C8	0.0278 (15)	0.0261 (15)	0.0222 (15)	0.0066 (12)	0.0050 (12)	0.0020 (12)
N1	0.0325 (14)	0.0270 (14)	0.0250 (13)	−0.0020 (11)	0.0004 (10)	0.0035 (10)
N2	0.0417 (15)	0.0376 (16)	0.0256 (14)	0.0011 (13)	−0.0005 (11)	−0.0016 (11)
N3	0.0368 (15)	0.0339 (15)	0.0314 (14)	0.0013 (12)	−0.0060 (11)	0.0046 (11)
N4	0.0283 (13)	0.0260 (13)	0.0181 (12)	−0.0002 (10)	0.0013 (10)	0.0006 (9)
N5	0.0254 (12)	0.0279 (14)	0.0204 (12)	0.0009 (10)	−0.0038 (10)	−0.0012 (10)
O1	0.0425 (13)	0.0377 (13)	0.0225 (11)	−0.0097 (10)	0.0000 (9)	−0.0058 (9)
O2	0.0404 (12)	0.0284 (12)	0.0328 (12)	−0.0072 (10)	0.0050 (9)	0.0021 (9)
O3	0.0446 (13)	0.0335 (13)	0.0268 (11)	−0.0012 (10)	0.0026 (10)	0.0074 (9)
O4	0.0393 (13)	0.0472 (14)	0.0207 (11)	0.0009 (10)	−0.0044 (9)	0.0056 (10)
O5	0.0620 (18)	0.068 (2)	0.0239 (14)	0.0243 (15)	0.0062 (12)	0.0123 (13)
Zn1	0.0372 (3)	0.0384 (3)	0.0165 (3)	−0.0026 (2)	−0.0028 (2)	0.0000 (2)

### Geometric parameters (Å, °)

**Table:**

C1—N2	1.322 (4)	C6—O2	1.265 (3)
C1—N3	1.353 (4)	C7—N5	1.371 (4)
C1—H1	0.9300	C7—C8	1.482 (4)
C2—N3	1.322 (4)	C8—O4	1.220 (3)
C2—N1	1.332 (4)	C8—O3	1.298 (3)
C2—H2	0.9300	N1—N2	1.363 (3)
C3—N1	1.459 (4)	N4—Zn1	2.110 (2)
C3—C4	1.491 (4)	N5—H5A	0.8600
C3—H3A	0.9700	O1—Zn1	2.115 (2)
C3—H3B	0.9700	O3—H3C	0.8500
C4—N4	1.325 (4)	O5—Zn1	2.154 (3)
C4—N5	1.337 (3)	O5—H5B	0.73 (5)
C5—C7	1.370 (4)	O5—H5C	0.78 (5)
C5—N4	1.377 (3)	Zn1—N4^i^	2.110 (2)
C5—C6	1.482 (4)	Zn1—O1^i^	2.115 (2)
C6—O1	1.252 (3)	Zn1—O5^i^	2.154 (3)
			
N2—C1—N3	114.9 (3)	N2—N1—C3	122.6 (2)
N2—C1—H1	122.6	C1—N2—N1	102.2 (2)
N3—C1—H1	122.6	C2—N3—C1	102.7 (3)
N3—C2—N1	110.6 (3)	C4—N4—C5	105.7 (2)
N3—C2—H2	124.7	C4—N4—Zn1	144.30 (19)
N1—C2—H2	124.7	C5—N4—Zn1	109.97 (18)
N1—C3—C4	112.2 (2)	C4—N5—C7	107.9 (2)
N1—C3—H3A	109.2	C4—N5—H5A	126.1
C4—C3—H3A	109.2	C7—N5—H5A	126.1
N1—C3—H3B	109.2	C6—O1—Zn1	115.28 (18)
C4—C3—H3B	109.2	C8—O3—H3C	115.0
H3A—C3—H3B	107.9	Zn1—O5—H5B	115 (4)
N4—C4—N5	111.3 (2)	Zn1—O5—H5C	121 (4)
N4—C4—C3	124.7 (2)	H5B—O5—H5C	114 (5)
N5—C4—C3	124.0 (3)	N4—Zn1—N4^i^	180.000 (1)
C7—C5—N4	109.3 (2)	N4—Zn1—O1^i^	100.52 (8)
C7—C5—C6	132.5 (3)	N4^i^—Zn1—O1^i^	79.48 (8)
N4—C5—C6	118.3 (2)	N4—Zn1—O1	79.48 (8)
O1—C6—O2	125.1 (3)	N4^i^—Zn1—O1	100.52 (8)
O1—C6—C5	116.8 (3)	O1^i^—Zn1—O1	180.00 (8)
O2—C6—C5	118.1 (3)	N4—Zn1—O5^i^	90.04 (10)
C5—C7—N5	105.8 (2)	N4^i^—Zn1—O5^i^	89.96 (10)
C5—C7—C8	132.6 (3)	O1^i^—Zn1—O5^i^	90.32 (11)
N5—C7—C8	121.6 (2)	O1—Zn1—O5^i^	89.68 (11)
O4—C8—O3	123.1 (3)	N4—Zn1—O5	89.96 (10)
O4—C8—C7	120.4 (3)	N4^i^—Zn1—O5	90.04 (10)
O3—C8—C7	116.6 (3)	O1^i^—Zn1—O5	89.68 (11)
C2—N1—N2	109.5 (3)	O1—Zn1—O5	90.32 (11)
C2—N1—C3	127.8 (3)	O5^i^—Zn1—O5	180.000 (1)
			
N1—C3—C4—N4	−76.1 (4)	C3—C4—N4—Zn1	−3.2 (5)
N1—C3—C4—N5	102.9 (3)	C7—C5—N4—C4	1.0 (3)
C7—C5—C6—O1	−178.8 (3)	C6—C5—N4—C4	−177.7 (2)
N4—C5—C6—O1	−0.6 (4)	C7—C5—N4—Zn1	−178.12 (17)
C7—C5—C6—O2	1.6 (5)	C6—C5—N4—Zn1	3.2 (3)
N4—C5—C6—O2	179.9 (2)	N4—C4—N5—C7	0.3 (3)
N4—C5—C7—N5	−0.8 (3)	C3—C4—N5—C7	−178.8 (3)
C6—C5—C7—N5	177.6 (3)	C5—C7—N5—C4	0.3 (3)
N4—C5—C7—C8	177.3 (3)	C8—C7—N5—C4	−178.0 (2)
C6—C5—C7—C8	−4.3 (5)	O2—C6—O1—Zn1	177.0 (2)
C5—C7—C8—O4	−175.5 (3)	C5—C6—O1—Zn1	−2.5 (3)
N5—C7—C8—O4	2.3 (4)	C4—N4—Zn1—N4^i^	−159 (100)
C5—C7—C8—O3	4.4 (5)	C5—N4—Zn1—N4^i^	20 (100)
N5—C7—C8—O3	−177.8 (2)	C4—N4—Zn1—O1^i^	−1.8 (3)
N3—C2—N1—N2	−0.6 (3)	C5—N4—Zn1—O1^i^	176.69 (17)
N3—C2—N1—C3	−179.2 (3)	C4—N4—Zn1—O1	178.2 (3)
C4—C3—N1—C2	99.6 (3)	C5—N4—Zn1—O1	−3.31 (17)
C4—C3—N1—N2	−78.8 (3)	C4—N4—Zn1—O5^i^	88.5 (3)
N3—C1—N2—N1	0.5 (4)	C5—N4—Zn1—O5^i^	−93.0 (2)
C2—N1—N2—C1	0.1 (3)	C4—N4—Zn1—O5	−91.5 (3)
C3—N1—N2—C1	178.8 (3)	C5—N4—Zn1—O5	87.0 (2)
N1—C2—N3—C1	0.9 (3)	C6—O1—Zn1—N4	3.3 (2)
N2—C1—N3—C2	−0.9 (4)	C6—O1—Zn1—N4^i^	−176.7 (2)
N5—C4—N4—C5	−0.8 (3)	C6—O1—Zn1—O1^i^	−136 (100)
C3—C4—N4—C5	178.3 (3)	C6—O1—Zn1—O5^i^	93.4 (2)
N5—C4—N4—Zn1	177.7 (2)	C6—O1—Zn1—O5	−86.6 (2)

Symmetry codes: (i) −*x*+1, −*y*+2, −*z*+2.

### Hydrogen-bond geometry (Å, °)

**Table:**

*D*—H···*A*	*D*—H	H···*A*	*D*···*A*	*D*—H···*A*
N5—H5A···N3^ii^	0.86	1.95	2.801 (4)	170
O3—H3C···O2	0.85	1.65	2.493 (3)	173
O5—H5B···O4^iii^	0.73 (5)	2.04 (5)	2.764 (4)	168 (5)
O5—H5C···N2^iv^	0.78 (5)	2.23 (5)	2.896 (4)	143 (5)

Symmetry codes: (ii) *x*−1/2, −*y*+3/2, *z*−1/2; (iii) *x*, *y*, *z*+1; (iv) −*x*+1/2, *y*+1/2, −*z*+3/2.
